# A Case-Based Review of Advanced Surgical Techniques in the Treatment of Incidentally Diagnosed Fibrolamellar Hepatocellular Carcinoma

**DOI:** 10.7759/cureus.101612

**Published:** 2026-01-15

**Authors:** Truman Archer, Omar Barakat, Basem Soliman, Ahmed Elfadaly, Mohamed Elfedaly

**Affiliations:** 1 Department of Surgery, Texas Tech University Health Sciences Center, Amarillo, USA; 2 Department of Surgery, San Joaquin General Hospital, French Camp, USA

**Keywords:** fibrolamellar hepatocellular carcinoma, hepatectomy, hepatocellular carcinoma, incidentaloma, surgical technique

## Abstract

Fibrolamellar hepatocellular carcinoma (FL-HCC) is a rare liver tumor that typically affects young adults without underlying liver disease. This case describes an incidental diagnosis of FL-HCC in a healthy 25-year-old male who presented to the Emergency Department with left flank pain. Imaging revealed a ureteropelvic junction stone with moderate hydronephrosis, as well as an unexpectedly large right hepatic mass. Further evaluation confirmed the diagnosis of FL-HCC. The patient underwent a successful right hepatectomy utilizing the anterior approach with hanging maneuver and extra-Glissonian right pedicle control. Postoperatively, the patient experienced an uneventful recovery.

This case highlights the importance of thorough evaluation of incidental findings, even in asymptomatic and healthy individuals, and demonstrates the advantages of advanced surgical techniques in managing large liver tumors.

## Introduction

Fibrolamellar hepatocellular carcinoma (FL-HCC) is a rare subtype of HCC that occurs predominantly in adolescents and young adults without chronic liver disease or cirrhosis. It accounts for approximately 0.41% of all HCC diagnoses in the United States [1]. Unlike conventional HCC, FL-HCC often presents in otherwise healthy individuals - without symptoms of cirrhosis and mass effect, such as weight loss, ascites, and jaundice. It is often discovered incidentally during imaging for unrelated complaints. Surgical resection remains the mainstay of treatment [2].

For large liver tumors, particularly those in the right lobe, the surgical approach is critical to achieving optimal outcomes. The anterior approach with hanging maneuver and extra-Glissonian pedicle control represents advanced techniques that can significantly improve surgical outcomes in these challenging cases [3,4]. These approaches help the surgeon to avoid damaging nearby structures, particularly blood vessels, reduce blood loss, and avoid unnecessary and lengthy dissection processes associated with other methods.

In this report, we discuss a case of an incidentally discovered FL-HCC tumor in a 25-year-old and its subsequent resection via the aforementioned surgical techniques.

## Case presentation

A 25-year-old previously healthy Hispanic male presented to the Emergency Department with a primary complaint of acute-onset, non-radiating left flank pain associated with mild nausea. The pain began several hours prior to presentation and was not accompanied by vomiting, fever, chills, or gastrointestinal symptoms. He had no significant past medical history, took no medications, and had no prior abdominal surgeries.

On examination, the patient was hemodynamically stable and afebrile. Laboratory studies, including white blood cell count, renal panel, liver function tests, and pancreatic enzymes, were all within normal limits.

**Figure 1:**
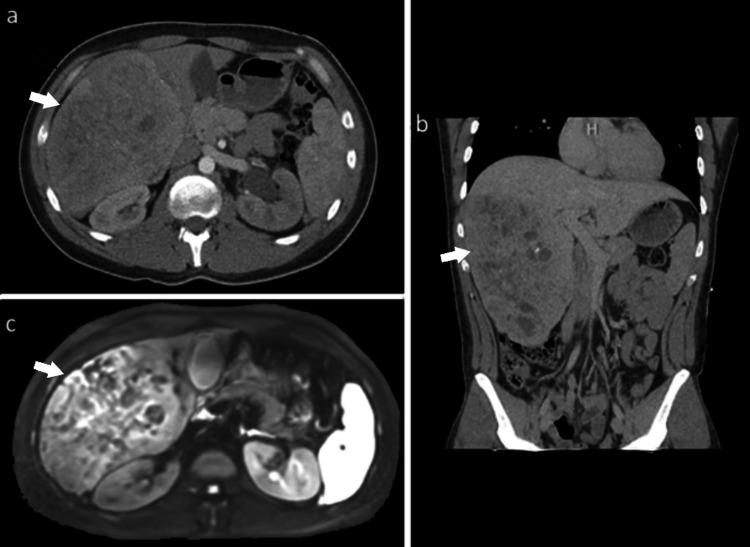
Pre-operative images a) Contrast CT - transverse, b) Contrast CT - coronal, c) T2-weighted transverse MRI. Subsequent imaging was obtained after a large right hepatic mass was discovered incidentally on CT evaluation for nephrolithiasis. Arrows point towards the right hepatic mass.

A non-contrast CT scan of the abdomen and pelvis was obtained, revealing a 5 mm obstructing left ureteropelvic junction stone with upstream moderate hydronephrosis. Incidentally, a large mass was noted in the right hepatic lobe (Figures 1a, 1b). The lesion was hypoattenuating on portal venous phase imaging and prompted further evaluation.

The patient denied right upper quadrant pain, weight loss, early satiety, or constitutional symptoms. Serum alpha-fetoprotein (AFP) level was within normal limits. Given the absence of underlying liver disease and the patient's demographic, fibrolamellar carcinoma was considered in the differential. A subsequent triple-phase liver MRI demonstrated a well-circumscribed, heterogeneous mass in the right lobe with features suspicious for HCC (Figure 1c).

**Figure 2:**
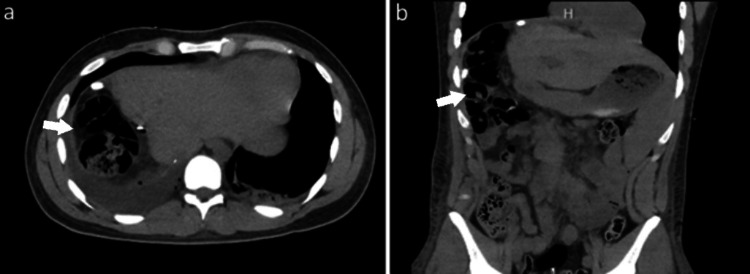
Post-operative images a) Contrast CT - transverse, b) Noncontrast CT - coronal. Arrows represent the site of operation for right hepatectomy.

The patient underwent a formal right hepatectomy without complication. Postoperative imaging is shown in Figure 2. He was extubated in the operating room and transferred to the surgical ICU for overnight monitoring. By postoperative day 1, he was stepped down to the floor. His recovery was unremarkable, and he was discharged on postoperative day 5. At his follow-up visits, he had returned to normal activity and had no signs of recurrence.

**Figure 3:**
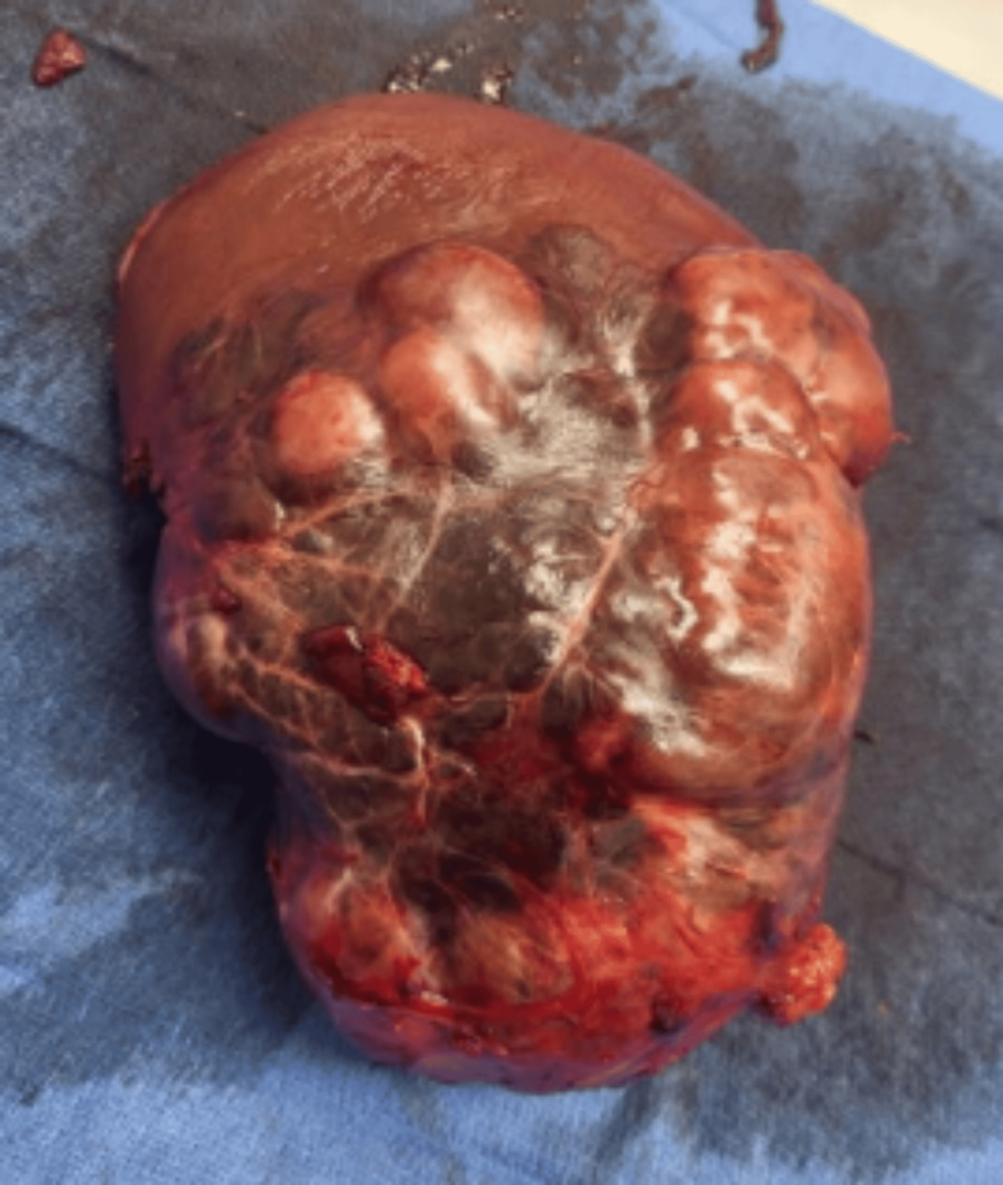
Gross specimen

Final histopathology described the mass as a 2349.2 g, 21.8 × 19.0 × 10.8 cm segment of liver with a 16.5 × 12.2 cm tan-red, soft, nodular capsule. The mass contained a 5.2 × 3.2 × 3.2 cm central cyst bordered by calcified change. The mass was noted to have negative margins and positive lymphovascular invasion. Gross appearance showed a lobulated appearance and microscopic evaluation of the well-differentiated mass revealed fibrous stroma with parallel collagen fibers separating tumor trabeculae, as shown in Figure 3. Many large polygonal tumor cells demonstrated vesicular nuclei with prominent nucleoli and abundant eosinophilic cytoplasm. The gross appearance, microscopic features, and background of non-cirrhotic parenchyma were highly suggestive of FL-HCC.

## Discussion

Epidemiology and diagnosis of FL-HCC

FL-HCC is a distinct clinical and pathological entity, typically occurring in individuals aged 10-35 years [1]. Unlike conventional HCC, it arises in non-cirrhotic livers and is not associated with common risk factors such as hepatitis B or C infection, alcohol use, or metabolic syndrome. The estimated incidence of FL-HCC is 0.02 per 100,000 in the U.S., with no clear gender predominance [5]. The etiology remains unclear, though recent studies have identified a characteristic DNAJB1-PRKACA fusion gene, resulting from a ~400 kb deletion on chromosome 19, occurring in many cases [6].

Imaging features on CT and MRI include a large, heterogeneous mass, often containing a central scar and calcifications. However, AFP is usually normal, as seen in this patient. Diagnosis is confirmed by histopathological evaluation, demonstrating large polygonal cells with eosinophilic cytoplasm, large nuclei, and fibrous lamellar bands.

Surgical rationale and advantages of the anterior approach

The patient underwent a formal right hepatectomy. Given the tumor's large size and its location in the right hepatic lobe, an anterior approach without liver mobilization was utilized. The transection plane was followed precisely after performing the hanging maneuver. A nasogastric tube was passed between the anterior surface of the inferior vena cava (IVC) and the liver inferiorly, and retrieved in the dissected plane between the right hepatic vein (RHV) and the middle hepatic vein (MHV).

The anterior approach for right hepatectomy, particularly for large tumors, offers several significant advantages over the conventional approach. Liu et al. conducted a prospective randomized controlled study on 120 patients with large HCC and found that the anterior approach resulted in better operative and survival outcomes compared with the conventional approach [7]. The anterior approach was associated with significantly less major operative blood loss (≥2 L), occurring in only 8.3% of cases compared to 28.3% in the conventional approach group (p = 0.005). This resulted in lower blood transfusion requirements and fewer patients requiring blood transfusion. More importantly, overall survival was significantly better in the anterior approach group (median > 68.1 months) than in the conventional approach group (median = 22.6 months, p = 0.006) [7].

The anterior approach was chosen in our case due to the risk of tumor rupture with lateral liver mobilization. This approach avoids several complications associated with conventional liver mobilization. First, excessive bleeding from avulsion of hepatic veins during mobilization is reduced [8,9]. Prolonged ischemia of the liver remnant from rotation of the hepatoduodenal ligament is avoided [8]. There is a decreased incidence of iatrogenic tumor rupture and potential tumor spillage [7,10]. This limits systemic dissemination of cancer cells into the circulation due to tumor manipulation [11]. Finally, this approach reduces risk of hemodynamic instability from IVC compression during right liver mobilization, particularly in cases involving large tumors [8].

Hanging maneuver and extra-Glissonian control

The hanging maneuver, first described by Belghiti et al. in 2001, significantly enhances the safety and efficacy of the anterior approach [3]. This technique involves passing a tape or nasogastric tube between the anterior surface of the IVC and the posterior surface of the liver, allowing the liver to be suspended during parenchymal transection. Fleres et al. described the technique as providing several key advantages, including guidance of the direction of anatomic parenchymal transection along the shortest and most anatomically correct route [12]. This results in maintenance of optimal orientation in conditions with distorted anatomy, such as with large tumors, as well as reduction of venous backflow bleeding through upward traction similar to digital compression. Furthermore, the facilitation of exposure of the deeper parenchymal plane allows better visualization and control of bleeding vessels. Lastly, the hanging approach eliminates the need for wide mobilization of the right liver [13].

A study by Makdissi et al. demonstrated that the hanging maneuver allows for straightforward access and control of the main Glissonian pedicle without time-consuming dissection of the hilar structures [13]. This is particularly valuable in cases with large tumors where conventional hilar dissection may be technically challenging. The extra-Glissonian approach offers several advantages over conventional hilar dissection. Giordano et al. demonstrated comparable operative times between pedicle stapling using the extra-Glissonian approach and conventional hilar dissection (240 vs. 260 min; p = 0.230), despite the extra-Glissonian approach allowing for en bloc control of all hilar structures without individual dissection [14]. The same study also found no significant differences in hemorrhage (800 mL vs. 730 mL, p = 0.699), transfusion requirements, or major complications (grade ≥3a) between the extra-Glissonian and conventional approaches. Mortality rates were also similar (4.9% vs. 3.5%; p = 0.882) [14].

The extra-Glissonian approach minimizes the risk of iatrogenic injury to left-sided structures, as demonstrated by Mouly et al., who noted that this approach allows for selective vascular inflow control while protecting the future liver remnant from ischemia-reperfusion injury [15]. Studies have shown that the extra-Glissonian approach allows for accurate determination of the anatomic liver area to be resected, particularly important in anatomical resections for HCC [16]. In selective clamping applications, the extra-Glissonian approach avoids ischemia to the contralateral hemiliver, which is particularly important in patients with underlying liver disease [14]. Selective clamping with the extra-Glissonian approach prevents splanchnic stasis and requires lower fluid replacement, contributing to improved hemodynamic stability during the procedure [14].

**Figure 4:**
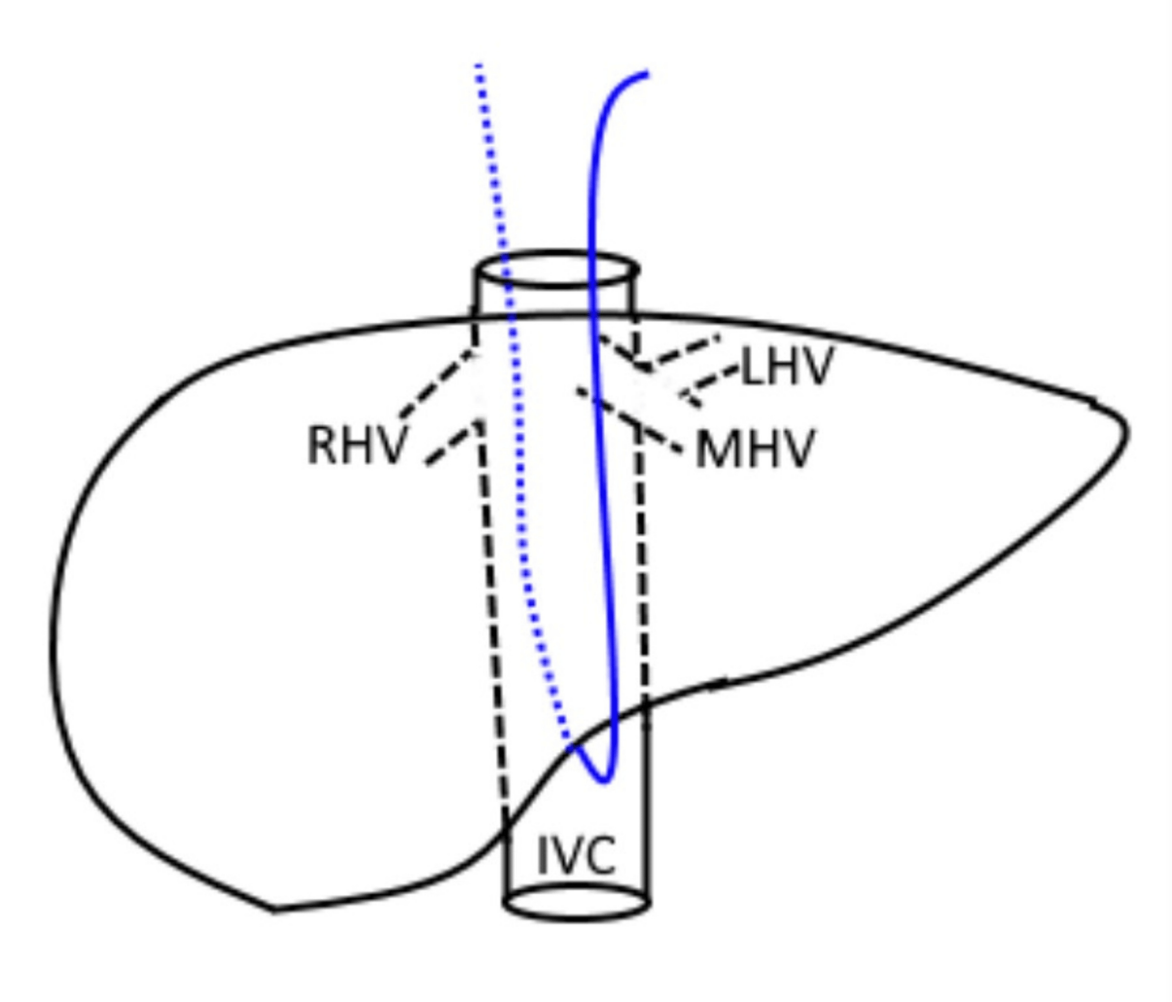
Hanging maneuver Credit: Created by the authors The nasogastric tube (blue) is run between the RHV and the MHV, and then used to lift the liver. RHV, right hepatic vein; MHV, middle hepatic vein; LHV, left hepatic vein; IVC, inferior vena cava

The hanging maneuver (Figure 4) was performed following the key surgical steps described by Belghiti et al. and refined by Fleres et al. [3,12]. The space between the RHV and the MHV was exposed by extending the falciform ligament division superiorly and opening the cranial right coronary ligament. This space was dissected downwards along the vena cava axis for 3-4 cm using a curved dissector with gentle movements. Next, a small opening was created at the intersection between the anterior aspect of the infra-hepatic vena cava and the caudate lobe, accessing the virtual space between the vena cava adventitia and the liver capsule (Laennec's capsule). A nasogastric tube was then passed from the lower dissection area, along the avascular plane of the retrohepatic IVC (at the 10-11 o'clock position), and retrieved in the previously dissected space between the RHV and MHV. Lastly, the nasogastric tube was used to lift the liver during parenchymal transection, providing both a reduction of venous backflow bleeding and guidance for the transection plane.

**Figure 5:**
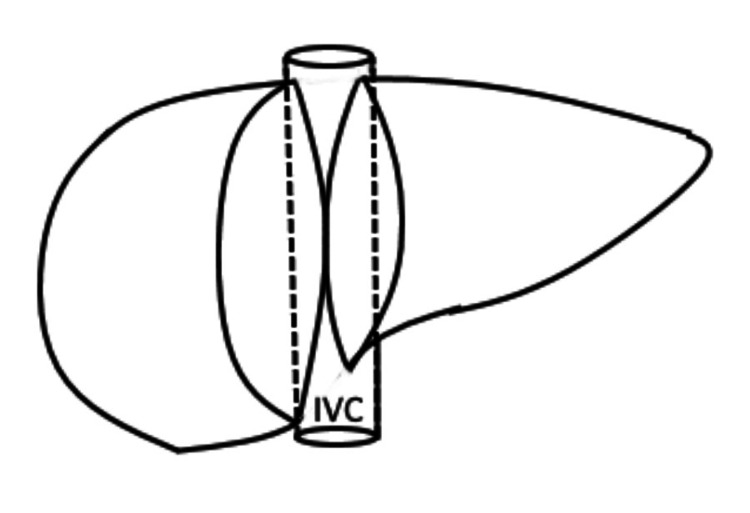
Anterior approach Credit: Created by the authors The liver is transected from the anterior side to the IVC. IVC, inferior vena cava

The anterior approach (Figure 5) for liver transection was performed as described by Liu et al. [7]. The right liver and tumor were not mobilized at the beginning of the procedure. Instead, hilar dissection was begun directly. After hilar dissection, the plane of parenchymal transection was marked on Glisson's capsule with the help of intraoperative ultrasonography. The transection was then performed along the marked plane using an ultrasonic dissector, without prior mobilization of the right liver. The hanging maneuver was then applied: the nasogastric tube, placed between the anterior surface of the IVC and the liver, was used to lift the liver during transection, facilitating exposure and bleeding control. Only after completion of parenchymal transection was the right liver mobilized from the retroperitoneum. The short hepatic veins were individually isolated, ligated, and transected after liver transection was complete.

**Figure 6:**
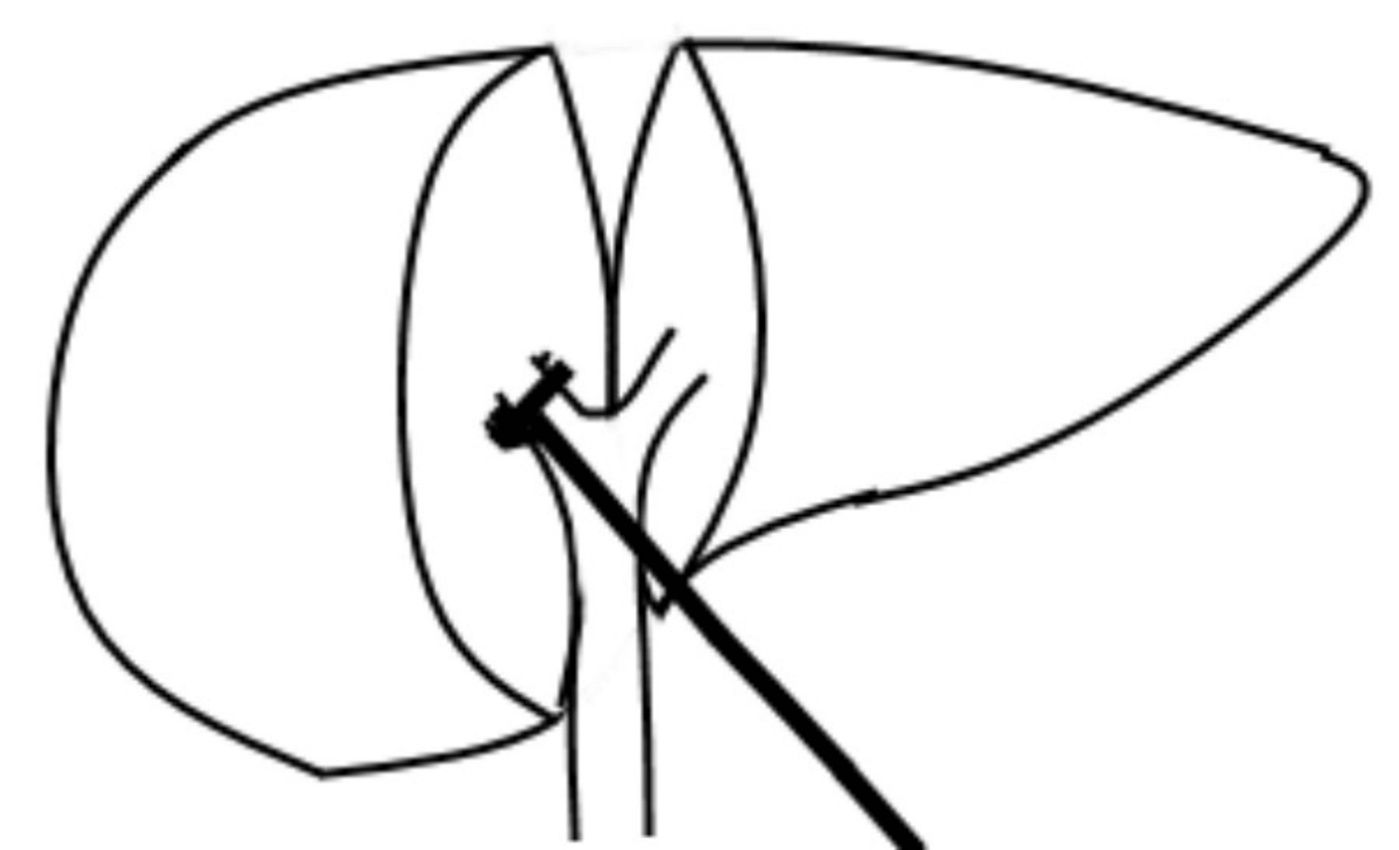
Extra-Glissonian approach Credit: Created by the authors The right hepatic pedicle was isolated and transected en bloc using the TA-30 vascular stapler (white type).

The extra-Glissonian approach for right hepatectomy (Figure 6) was performed following the key surgical steps described by Takasaki et al. and refined by Giordano et al. [14,17]. The hilar plate was lowered at the base of Segment IV of the liver to expose the entry point for the extra-Glissonian approach. A large right-angle clamp was passed from the entry point toward the exit point, just inferior to the right posterior portal pedicle at the sulcus of Rouvière. A vascular stapler (TA-30, white type) was introduced to transect en bloc the right portal triad, including the portal vein, hepatic artery, and biliary duct, all contained within Glisson's sheath. During pedicle transection, countertraction of the loop was performed to avoid injury to the bile duct confluence and contralateral structures. After clamping, the ischemic demarcation line was confirmed before proceeding with parenchymal transection.

Surgical resection remains the cornerstone of treatment for FL-HCC and offers the best chance of long-term survival, especially in the absence of metastases. Five-year survival rates after complete resection range from 40% to 70%, depending on tumor burden, nodal involvement, and resectability [18]. The role of chemotherapy and radiotherapy remains limited and is generally considered in unresectable or metastatic cases.

Prognosis and general principles

In conclusion, this case highlights the importance of thorough investigation of incidental findings in imaging, even when obtained for unrelated complaints. In young, healthy patients without liver disease, the identification of a liver mass should prompt consideration of fibrolamellar carcinoma. Early detection and surgical intervention are critical to improving outcomes.

## Conclusions

The successful management of this case demonstrates the significant advantages of utilizing the anterior approach with hanging maneuver and extra-Glissonian pedicle control for right hepatectomy in the setting of a large liver tumor. These techniques offer improved safety, reduced blood loss, better visualization during parenchymal transection, and potentially improved long-term outcomes, compared to conventional approaches. The standardization of these techniques provides a valuable framework for hepatobiliary surgeons managing similar, challenging cases.

Beyond the technical success involved, this case shows the critical importance of careful evaluation of incidental radiographic findings, particularly in young patients without obvious hepatic pathology. FL-HCC, although rare, should remain in the differential diagnosis for large hepatic lesions identified in otherwise healthy individuals. Early recognition and application of advanced surgical strategies optimize perioperative outcomes and improve survival in this patient population. As surgical innovations continue to evolve, broader dissemination and training in new approaches are essential for ensuring reproducibility and improving access to safe, effective resection techniques in complex hepatobiliary surgery.
